# Optimizing Traumatic Brain Injury Care Without Neurosurgeons: External Validation of the Brain Injury Guidelines in a Resource-Limited Trauma System

**DOI:** 10.3390/jcm15114262

**Published:** 2026-05-31

**Authors:** Stéphanie Santin, Bellal Joseph, Rafael Dib Possiedi, Leticia Stefani Pacheco, Lara Portugal De Santana, Christina Maria Rossiter Wade, Marcelo Augusto Fontenelle Ribeiro

**Affiliations:** 1 Graduate Program in Health Sciences, Medical Assistance Institute for State Public Servants (IAMSPE), São Paulo 04029000, São Paulo, Brazil; stephaniesantin21@gmail.com; 2 Grady Memorial Hospital, Atlanta, GA 30303, USA; bjoseph2@gmh.edu; 3 R Adams Cowley Shock Trauma Center, School of Medicine, University of Maryland, Baltimore, MD 21201, USA; rpossiedi@som.umaryland.edu; 4 School of Medicine, Pontifical Catholic University of São Paulo, Sorocaba 18030070, São Paulo, Brazil; ra00298473@pucsp.edu.br; 5 Bahiana School of Medicine and Public Health (EBMSP), Salvador 40290000, Bahia, Brazil; larasantana23.2@bahiana.edu.br; 6 Rutgers New Jersey Medical School, Newark, NJ 07103, USA; cw1064@njms.rutgers.edu

**Keywords:** traumatic brain injury, brain injury guidelines, acute care surgery, neurosurgical consultation, resource-limited setting

## Abstract

**Background/Objectives**: Access to neurosurgical care remains limited in many trauma systems worldwide, particularly in low- and middle-income countries (LMICs). The Brain Injury Guidelines (BIG) were developed to guide the management of traumatic brain injury (TBI) and optimize resource utilization; however, their applicability in resource-limited environments without on-site neurosurgical coverage remains unclear. The aim of this study was to evaluate the performance and applicability of the BIG in a trauma center without neurosurgical support. **Methods**: We performed a retrospective analysis of adult patients with TBI admitted to a trauma center without neurosurgical support in São Paulo, Brazil, between 2013 and 2017. Patients were classified according to the BIG criteria (BIG 1–3) based on clinical and radiological findings. Primary outcomes were clinical and radiological deterioration and mortality; secondary outcomes included neurosurgical transfer, repeat computed tomography (CT) utilization, and length of stay. **Results**: A total of 178 patients were included: 12 (6.7%) BIG 1, 53 (29.8%) BIG 2, and 113 (63.5%) BIG 3. No patient classified as BIG 1 or BIG 2 experienced clinical or radiological deterioration, required neurosurgical intervention, or died; adverse outcomes were confined to the BIG 3 cohort, with a mortality rate of 11.5%. The combined BIG 1–2 group showed a sensitivity and negative predictive value (NPV) of 100% for identifying patients without deterioration or need for neurosurgical intervention. Despite the absence of adverse events in the BIG 1–2 group, 76.4% of patients underwent transfer for neurosurgical evaluation, and repeated CT imaging was frequently performed. **Conclusions**: In this single-center retrospective cohort, the BIG demonstrated excellent discriminatory ability for identifying low-risk TBI patients in a setting without neurosurgical coverage. BIG 1 and BIG 2 categories reliably ruled out the need for neurosurgical intervention, supporting selective non-transfer strategies to optimize resource utilization.

## 1. Introduction

Traumatic brain injury (TBI) remains a leading cause of morbidity and mortality worldwide, with an estimated 50 million new cases annually and a disproportionate burden in low- and middle-income countries (LMICs) [1,2,3]. Beyond its clinical impact, TBI imposes substantial socioeconomic costs due to long-term disability and loss of productivity. Despite advances in critical care and imaging, the early management of TBI continues to rely heavily on timely diagnosis, appropriate triage, and access to specialized care. A central challenge in many trauma systems, especially in LMICs, is the limited availability of neurosurgical services [4].

In several regions, the initial evaluation and management of TBI are performed by general or trauma surgeons, with neurosurgical consultation requiring interfacility transfer. This model often leads to over-triage, with a large proportion of patients transferred for specialist evaluation despite ultimately requiring no intervention. In Brazil, this challenge is compounded by the unequal geographic distribution of specialists [5].

The widespread use of computed tomography (CT) has improved the detection of intracranial injuries, particularly in mild TBI [6,7]. Traditional classification systems such as the Glasgow Coma Scale (GCS) provide important clinical information but do not incorporate radiological findings and therefore offer limited guidance for resource utilization [8]. The Brain Injury Guidelines (BIG) were developed to address these limitations by integrating clinical and radiological parameters into a structured management algorithm [9]. Patients are stratified into three categories (BIG 1–3), with corresponding recommendations regarding observation, repeat imaging, hospitalization, and neurosurgical consultation. Initial studies have demonstrated that the BIG can safely reduce unnecessary neurosurgical consultations, repeat CT scans, and hospital admissions without compromising patient outcomes [10,11].

However, the applicability of the BIG in resource-limited environments, particularly in centers without on-site neurosurgical coverage, remains insufficiently explored. The aim of this study was to evaluate the performance and applicability of the Brain Injury Guidelines in a trauma center without neurosurgical support.

## 2. Materials and Methods

### 2.1. Study Design and Patient Population

This was a retrospective study analyzing data obtained from the hospital electronic medical record system (Tasy®, version 2.2.x; Philips Healthcare, Eindhoven, The Netherlands) from all patients with a diagnosis of TBI admitted between December 2013 and April 2017 to Grajaú General Hospital, in the city of São Paulo, Brazil—a public trauma center without an on-site neurosurgical team.

### 2.2. Inclusion and Exclusion Criteria

Data were obtained from the electronic medical records of patients aged 13 years or older, regardless of trauma mechanism. Patients with positive findings on the initial head CT scan, as reported in the radiology report, were included. Patients with severe associated trauma or missing data were excluded.

### 2.3. Outcome Measures

The outcomes measured were the rate of repeat head CT scans, the rate of transfer for neurosurgical evaluation and intervention, the rate of clinical and radiological deterioration, and mortality.

### 2.4. Variables Assessed

Categorical and numerical variables included sex, age, length of stay, systolic blood pressure (SBP) and heart rate (HR), trauma mechanism, neurological alterations (focal deficit and pupillary abnormalities), pre-injury use of anticoagulant and/or antiplatelet medications, and the GCS score on admission and at 24 h. Additional variables included the presence of blunt injury to the head, CT findings, follow-up (control) CT results, number of CT scans performed during hospitalization, need for intensive care unit (ICU) admission, neurosurgical assessment, and outcome.

The following CT scan findings were considered: skull fracture with or without displacement, subdural hematoma (SDH), extradural hematoma (EDH), intraparenchymal hemorrhage (IPH), subarachnoid hemorrhage (SAH), and intraventricular hemorrhage (IVH). In patients who underwent follow-up CT scans, images were re-evaluated to determine whether lesions remained unchanged, increased, or decreased in size, and whether any new lesions had appeared.

Trauma mechanisms were classified as low-energy (ground-level falls and assaults), high-energy (motor vehicle collisions, motorcycle crashes, pedestrian strikes, and falls from >3 m), and other (falls from a bed, chair, bicycle, or skateboard).

When a neurosurgical evaluation was required, patients were transferred to another facility according to the allocation provided by the São Paulo municipal on-call coordination system. Because of the lack of an integrated electronic health record connecting public hospitals in São Paulo, the medical records of patients transferred for neurosurgical treatment were not accessible for follow-up. Patients who returned to the referring hospital were treated conservatively until discharge.

### 2.5. The Brain Injury Guidelines

After data collection, patients were retrospectively classified as BIG 1, BIG 2, or BIG 3 according to the criteria proposed by Joseph et al. [9]. Classification was performed by trained study investigators who reviewed the radiology reports and clinical records, with cases of uncertainty resolved by consensus with the senior author. The BIG classification system is summarized in Table 1.

An abnormal neurological examination was defined as a focal neurological deficit, a pupillary abnormality, or a GCS score of 12 or less. Patients with a GCS < 15 and an abnormal neurological examination were categorized as BIG 3. If more than one lesion type was identified on CT imaging, or if clinical deterioration occurred, the patient was classified as BIG 3 regardless of lesion size. Because toxicological testing was not routinely performed, any neurological alteration present was assumed to result from the trauma rather than from intoxication.

Patients classified as BIG 1 do not require neurosurgical assessment or a repeat CT scan; management includes neurological observation for 6 h, followed by hospital discharge (if no neurological change occurs) and outpatient follow-up. BIG 2 patients require hospital admission but no neurosurgical assessment or repeat head CT. BIG 3 patients require hospital admission, neurosurgical assessment, and a repeat head CT [9].

### 2.6. Statistical Analysis

Categorical variables were described using frequencies and percentages, and continuous variables using means, medians, and standard deviations. Comparisons between BIG groups were performed using the chi-square test for categorical variables and the Kruskal–Wallis test for continuous variables. Normality was assessed with the Shapiro–Wilk test. A significance level of 5% was adopted. Analyses were performed using STATA version 10.0 (StataCorp LLC, College Station, TX, USA).

### 2.7. Diagnostic Performance Analysis

To evaluate the ability of the BIG to identify patients at risk of adverse outcomes, patients were grouped into low-risk (BIG 1–2) and high-risk (BIG 3) categories. The primary outcome was a composite of clinical deterioration, radiological worsening, neurosurgical intervention, or mortality. Sensitivity and negative predictive value (NPV) were calculated based on the available in-hospital data. Given the incomplete follow-up of transferred patients, these estimates were considered exploratory. To assess the robustness of the diagnostic estimates to incomplete follow-up, a worst-case sensitivity analysis was performed in which every patient who was definitively transferred to another institution, and therefore lost to follow-up, was conservatively assumed to have experienced the composite adverse outcome; the sensitivity and NPV of the BIG 1–2 stratum were then recalculated under this assumption. In addition, a secondary analysis was conducted in which intensive care unit (ICU) admission was excluded from the composite outcome, in order to determine whether the diagnostic performance of the BIG was being driven by institutional admission practices rather than by objective clinical or radiological deterioration.

Artificial intelligence tools were used during manuscript preparation to assist with citation management (Zotero, https://www.zotero.org, accessed on 25 May 2026), study screening (Rayyan, https://www.rayyan.ai, accessed on 25 May 2026), academic writing support and plagiarism checking (Ref-N-Write, https://www.ref-n-write.com, accessed on 25 May 2026), and language editing (ChatGPT, GPT-5.5, OpenAI, San Francisco, CA, USA; https://chatgpt.com, accessed on 25 May 2026). The authors reviewed and approved all AI-assisted content and are fully responsible for its accuracy and integrity.

## 3. Results

A total of 195 patients with TBI were admitted during the study period. Seventeen patients were excluded due to missing data, leaving 178 patients for analysis. According to the BIG classification, 12 patients (6.7%) were classified as BIG 1, 53 (29.8%) as BIG 2, and 113 (63.5%) as BIG 3. Baseline demographic and clinical characteristics are summarized in Table 2.

Most patients presented with mild TBI, with 155 patients showing a GCS score between 13 and 15. Of the 178 patients, 122 patients maintained the same GCS score throughout their assessment, and 47 showed improvement. Nine patients showed neurological worsening (GCS changes: 6 → 3, 7 → 3 in two patients, 7 → 5, 8 → 3, 11 → 6, 12 → 5, 13 → 8, and 15 → 14). Abnormal neurological findings were identified in 9% of cases. Regarding hemodynamic status, 97.2% of patients had an SBP > 90 mmHg, and 23% had tachycardia. Blunt injuries were present in 36.3% of cases, and pre-injury anticoagulation was used by 2.2% of patients.

The most common trauma mechanisms were ground-level falls (32.6%) and falls from >3 m (25.8%). Traffic-related incidents (motor vehicle collisions, motorcycle crashes, and pedestrian strikes) accounted for 15.7%.

An initial CT scan was not performed in only one case, in which MRI was used instead due to equipment unavailability at the time. The average number of CT scans performed per patient during hospitalization was 2.2. Isolated SDH was the most frequent finding (18%), followed by isolated IPH (15%). Combined injuries were present in 22.5% of patients, with SAH plus contusion or IPH being the most frequent combination. Skull fractures were identified in 58.9% of patients. Among patients who underwent a repeat head CT scan, 29% showed improvement or resolution of findings.

The hospital length of stay ranged from 1 to 28 days (mean 5.09 days), and 4% of patients were admitted to the ICU. Most patients (76.4%) were transferred for neurosurgical evaluation, of whom only 2.3% were definitively transferred to another institution. The overall mortality rate was 7.3%.

Clinical outcomes and resource utilization by BIG classification are summarized in Table 3. No patient classified as BIG 1 or BIG 2 experienced clinical or radiological deterioration, required neurosurgical intervention, or died. Adverse outcomes occurred exclusively in the BIG 3 group.

Age distribution was similar across BIG categories, with mean ages of 45.3 years for BIG 1, 43.5 years for BIG 2, and 47.1 years for BIG 3. Female patients were less likely than males to present with a higher BIG classification: 41.7% of BIG 1 patients were female, compared with only 15.9% of BIG 3 patients (*p* < 0.05).

The mean admission GCS was lower in BIG 3 patients (13.1) than in BIG 1–2 patients (14.7) (*p* < 0.001). Hemodynamic parameters—heart rate, systolic blood pressure, and pre-injury anticoagulant use—were compared across groups. Ground-level falls were the most common mechanism across all BIG categories.

The number of repeat head CT scans increased with BIG category, with an average of 2.5 scans in BIG 3 compared with 1.7 in BIG 1 and BIG 2 combined (*p* < 0.001). No patient in BIG 1 or BIG 2 developed radiological worsening or new lesions. The number of skull fractures identified on CT was similar between BIG 2 and BIG 3. The median length of stay was 2.5 days for BIG 1, 3 days for BIG 2, and 4 days for BIG 3; the mean length of stay in BIG 3 was 5.9 days, significantly longer than in BIG 1 and BIG 2 (*p* < 0.05).

ICU admission occurred exclusively in BIG 3 patients (6.2%, 7/113). Rates of neurosurgical assessment and surgical treatment did not differ significantly between groups. The mortality rate in BIG 3 was 11.5%. Eight of these deaths resulted from TBI complications: 5 patients met brain death criteria, but 2 had the brain death protocol initiated but died before the second examination due to hydroelectrolyte disturbances, hypothermia, or shock. Five additional deaths were due to infection (one cutaneous and four respiratory). No deaths occurred in BIG 1 or BIG 2 patients. Overall mortality was 7.3%.

When patients were stratified into low-risk (BIG 1–2) and high-risk (BIG 3) groups, all adverse outcomes (clinical deterioration, radiological worsening, need for neurosurgical intervention, and mortality) occurred exclusively in the BIG 3 cohort. No patient classified as BIG 1 or BIG 2 experienced any of these outcomes (Table 4).

Based on these findings, the BIG classification demonstrated a sensitivity of 100% for identifying patients at risk of adverse outcomes in this cohort. Similarly, the NPV of the combined BIG 1–2 group was 100%, indicating that no low-risk patient experienced deterioration or required neurosurgical intervention.

Specificity could not be reliably estimated due to the high proportion of patients classified as BIG 3 and the absence of complete outcome data for all transferred patients. These results should therefore be interpreted with caution and regarded as exploratory. Diagnostic performance metrics are summarized in Table 5.

Two sensitivity analyses were performed to test the robustness of these estimates. First, loss to follow-up was confined to the small subset of patients who were definitively transferred to another institution for ongoing neurosurgical management (2.3% of transferred patients), all of whom belonged to the BIG 3 stratum; patients who returned to the referring hospital were followed until discharge. Even when each of these definitively transferred patients was conservatively assumed to have experienced the composite adverse outcome, the event rate within the BIG 3 group increased but the BIG 1–2 stratum was unaffected, and its sensitivity and NPV remained 100%. Second, when ICU admission was excluded from the composite outcome, all remaining adverse events (clinical deterioration, radiological worsening, neurosurgical intervention, and in-hospital mortality) again occurred exclusively in BIG 3 patients; the sensitivity and NPV of the BIG 1–2 stratum were therefore unchanged (100%), and only the positive predictive value within the BIG 3 group was modestly affected. Together, these analyses indicate that the discriminatory performance of the BIG for identifying low-risk patients was robust both to incomplete follow-up and to the inclusion of ICU admission in the composite outcome.

## 4. Discussion

This study evaluates the applicability of the BIG in a resource-limited trauma center without on-site neurosurgical coverage and demonstrates several clinically relevant findings. Most notably, patients classified as BIG 1 and BIG 2 experienced no clinical or radiological deterioration, required no neurosurgical intervention, and had no mortality. These findings translated into an NPV of 100% for adverse outcomes, reinforcing the potential safety of the BIG as a triage tool in environments where neurosurgical resources are not readily available. This perfect NPV is consistent with recent large systematic reviews and meta-analyses comprising thousands of patients, which have established a pooled sensitivity of 1.00 for the BIG criteria in predicting the need for neurosurgical intervention [12]. Further external validations in varied international and rural trauma centers have consistently replicated this safety profile for BIG 1 and BIG 2 cohorts [13,14].

The implications of these findings extend beyond diagnostic classification. Rather than serving solely as a clinical scoring system, the BIG function as a systems-level decision-making framework, guiding the allocation of critical resources such as neurosurgical consultation, interfacility transfer, and advanced imaging. In the present study, despite the absence of adverse outcomes in low-risk patients, more than three-quarters of patients were transferred for neurosurgical evaluation, and repeat CT imaging was frequently performed. Recent literature indicates that this persistent gap between evidence-based risk stratification and real-world practice is driven largely by provider discomfort, medicolegal concerns, and ingrained institutional admission pathways at both referring and receiving centers [15]. However, targeted protocols using the BIG criteria have shown that BIG 1 and BIG 2 patients without polytrauma rarely experience neurological decline and can safely avoid interhospital transfer [16].

To optimize applicability across diverse trauma systems, recent literature proposes significant modifications to the guidelines. A major area of revision addresses pre-injury antiplatelet and anticoagulant use. Under the original criteria, any antithrombotic use automatically upgrades patients to the BIG 3 category. However, recent extensive evaluations have shown that low-dose aspirin use in otherwise BIG 1 patients does not increase the risk of hemorrhage progression, neurosurgical intervention, or mortality, suggesting that these patients can safely avoid escalation to BIG 3 [17,18,19]. Similarly, omitting anticoagulation status entirely from the BIG criteria for lower-risk groups has been shown to safely reduce neurosurgical consultations by more than 50% without increasing mortality [20]. Conversely, clinicians must remain cautious: patients classified as BIG 3 strictly because of severe traumatic features (“BIG 3-solo”) often have higher rates of neurosurgical intervention and clinical deterioration than those escalated solely on the basis of their anticoagulation status [21]. It should be emphasized, however, that the present study applied the original BIG criteria and therefore cannot directly evaluate the performance of these proposed modifications. Dedicated validation of the modified BIG in low- and middle-income country trauma systems remains an important and unmet research need.

New clinical parameters are also emerging to inform escalation. The presence of a concomitant blunt cerebrovascular injury (BCVI) with a Biffl grade > 1 significantly predicts clinical progression in TBI patients across all BIG categories, warranting its formal integration into risk-stratification schemas [22]. Radiographic interpretation is also evolving; for instance, specific findings such as SAH and IPH without accompanying clinical decline have proven to be poor independent predictors of neurosurgical intervention, indicating potential areas to safely de-escalate monitoring [23]. Adjunctive treatments are being refined as well: early venous thromboembolism prophylaxis (≤24 h) is considered safe for BIG 1 and BIG 2 patients, significantly reducing the incidence of deep vein thrombosis without increasing intracranial hemorrhage progression or mortality [24].

The BIG is also being adapted for specific populations: dedicated criteria have improved the safety and efficiency of care in elderly [25] and pediatric [26,27,28,29] trauma patients, while institutional and rural adaptations have further optimized neurosurgical consultation, repeat imaging, and ICU utilization [30,31].

While previous BIG validation studies have been conducted in Level I and II trauma centers, the present study extends its applicability to lower-resource settings.

A further consideration specific to resource-limited settings concerns the classification of neurological findings. Because toxicological screening was not routinely available, any neurological alteration was attributed to trauma rather than to intoxication, reflecting a conservative, safety-oriented interpretation of the BIG. Under the original criteria, a neurological abnormality attributable to alcohol or drugs may allow a patient to remain in BIG 1 or BIG 2, whereas the inability to exclude intoxication shifts that same patient to BIG 3. The likely net effect is an upward migration toward the BIG 3 category and, consequently, an inflated transfer rate. This carries implications both for the internal validity of our classification and for the generalizability of our findings: in centers where toxicology is routinely performed, a proportion of patients classified here as BIG 3 might be reclassified into lower-risk categories, which would further increase the number of patients who could safely avoid transfer. This consideration therefore reinforces, rather than weakens, the central observation that a substantial proportion of TBI patients in this setting were over-triaged.

This study has several limitations that should be considered when interpreting its findings. First, its retrospective, single-center design and reliance on data extracted from electronic medical records constrain causal inference and may introduce selection and information bias as well as residual confounding. Second, the sample size was relatively small, and the BIG 1 subgroup in particular comprised only 12 patients (6.7%), which limits the statistical power to detect infrequent adverse events and precludes robust subgroup analyses, including for the small proportion of patients (2.2%) with pre-injury anticoagulant or antiplatelet use. Third, the absence of an interoperable electronic health record across the referral network meant that post-transfer outcomes were unavailable for the small subset of patients who were definitively transferred elsewhere; consequently, specificity could not be reliably estimated and the diagnostic performance metrics should be regarded as exploratory, although the worst-case sensitivity analysis indicated that the central finding was robust to this incomplete follow-up. Fourth, toxicological screening was not routinely performed, so the default attribution of neurological alterations to trauma may have introduced classification bias, as discussed above. Fifth, formal inter-rater reliability (e.g., Cohen’s kappa) for the retrospective BIG classification was not quantified. Finally, because the study reflects a specific regional referral system and resource-allocation model, its external validity is constrained, and generalization to other low- and middle-income country trauma systems should be made with caution. Prospective, multicenter studies with larger samples, particularly to strengthen the BIG 1 subgroup, are warranted to confirm these findings and to validate modified BIG criteria across diverse low- and middle-income country settings.

## 5. Conclusions

The BIG classification demonstrated excellent safety in low-risk patients (BIG 1–2), with no clinical or radiological deterioration observed. These findings support its potential role as a triage and resource-optimization tool, reducing unnecessary transfers and imaging. As the literature evolves, incorporating modifications to address specific factors —such as low-dose aspirin use, pediatric and elderly criteria, and BCVI integration—will be paramount to maximizing the clinical utility and safety of the Brain Injury Guidelines worldwide.

## Figures and Tables

**Table 1:** Brain Injury Guidelines (BIG) classification and management.

Variables	BIG 1	BIG 2	BIG 3
Loss of consciousness	Yes/No	Yes/No	Yes/No
Neurological examination	Normal	Normal	Abnormal
Intoxication	No	Yes/No	Yes/No
Oral anticoagulant, ASA	No	No	Yes
Skull fracture	No	Non-displaced	Displaced
Subdural hematoma	≤4 mm	5–7 mm	≥8 mm
Extradural hematoma	≤4 mm	5–7 mm	≥8 mm
Intraparenchymal hemorrhage	≤4 mm, 1 location	3–7 mm, 2 locations	≥8 mm, multiple locations
Subarachnoid hemorrhage	Trace	Localized	Scattered
Intraventricular hemorrhage	No	No	Yes
Therapeutic plan			
Hospitalization	No. Observation (6 h)	Yes	Yes
Follow-up head CT	No	No	Yes
Neurosurgical evaluation	No	No	Yes

Note. ASA = acetylsalicylic acid (aspirin); CT = computed tomography. An abnormal neurological examination was defined as a focal neurological deficit, a pupillary abnormality, or a Glasgow Coma Scale score ≤ 12. Importantly, if more than one lesion type was identified on the initial head CT, the patient was automatically classified as BIG 3 regardless of individual lesion size. Adapted from Joseph et al. [9].

**Table 2:** Baseline characteristics of the study population.

Variable	Category/Measure	*n*/Freq. (%)
**Demographics**		
Age (years)	Range	14–96
	Median	44
	Mean (SD)	45.9 (19.0)
	>60 years	39 (21.9)
Sex	Male	140 (78.7)
Glasgow Coma Scale (GCS)	13–15 (mild)	155 (87.1)
	9–12 (moderate)	9 (5.1)
	3–8 (severe)	14 (7.9)
Neurological findings	No abnormalities	162 (91.0)
	Pupillary abnormality	11 (6.2)
	Focal neurological deficit	4 (2.2)
	Pupillary + focal deficit	1 (0.6)
**Hemodynamic Status**		
Heart rate (bpm)	≥100	41 (23.0)
Systolic blood pressure (mmHg)	<90	5 (2.8)
Pre-injury anticoagulant/antiplatelet use	Yes	4 (2.2)
**Trauma Characteristics**		
Trauma mechanism	Ground-level fall	58 (32.6)
	Fall from height (>3 m)	46 (25.8)
	Motorcycle crash	14 (7.9)
	Pedestrian injury	13 (7.3)
	Assault	22 (12.4)
	Motor vehicle collision	1 (0.6)
	Other	24 (13.5)
**CT Scan Findings**		
Number of CT scans	Mean (SD)	2.2 (1.3)
CT–Intracranial hemorrhage	Subarachnoid hemorrhage (SAH)	24 (13.5)
	Subdural hematoma (SDH)	32 (17.9)
	Epidural hematoma (EDH)	24 (13.5)
	Intraparenchymal hemorrhage (IPH)/Contusion	27 (15.2)
	Intraventricular hemorrhage (IVH)	1 (0.6)
	No hemorrhage	22 (12.4)
	Combined injury	40 (22.5)
	Other	8 (4.5)
CT–Skull fracture ^a^	Non-displaced	81 (46.8)
	Displaced	21 (12.1)
	No fracture	71 (41.0)

Note. Values are presented as *n* (%) unless otherwise stated. SD = standard deviation; GCS = Glasgow Coma Scale; CT = computed tomography; SAH = subarachnoid hemorrhage; SDH = subdural hematoma; EDH = epidural hematoma; IPH = intraparenchymal hemorrhage; IVH = intraventricular hemorrhage. ^a^ Percentages for skull fracture calculated over patients with available CT data (*n* = 173; 5 patients with missing values excluded from denominator).

**Table 3:** Clinical outcomes and resource utilization by BIG classification.

Outcome	BIG 1 (*n* = 12)	BIG 2 (*n* = 53)	BIG 3 (*n* = 113)	*p*
Length of hospital stay, mean (SD), days	2.8 (1.9)	3.9 (3.5)	5.9 (5.5)	0.022
Length of hospital stay, median (range), days	2.5 (1–8)	3 (1–15)	4 (1–28)	
Number of CT scans, mean (SD)	1.7 (0.8)	1.7 (0.8)	2.5 (1.2)	<0.001
Repeat CT scan performed, *n* (%)	7 (58.3)	24 (45.3)	81 (71.7)	
Radiological improvement/resolution, *n* (%)	4 (33.3)	12 (22.6)	36 (31.9)	
Stable findings, *n* (%)	3 (25.0)	12 (22.6)	32 (28.3)	
Radiological worsening or new lesion, *n* (%)	0 (0)	0 (0)	13 (11.5)	
ICU admission, *n* (%)	0 (0)	0 (0)	7 (6.2)	0.12
Neurosurgical evaluation, *n* (%)	8 (66.7)	42 (79.2)	86 (76.1)	0.59
Neurosurgical intervention, *n* (%)	0 (0)	1 (1.9)	3 (2.7)	—
In-hospital mortality, *n* (%)	0 (0)	0 (0)	13 (11.5)	0.02

Note. BIG = Brain Injury Guidelines; CT = computed tomography; ICU = intensive care unit; SD = standard deviation. *p*-values derived from Kruskal–Wallis test (continuous variables) or Fisher’s exact test (categorical variables with low expected counts). Dashes (—) indicate metrics not tested due to zero-cell counts. Neurosurgical intervention rate is expressed as a proportion of the total group, not of those evaluated.

**Table 4:** Distribution of adverse outcomes according to BIG classification.

Adverse Outcome	BIG 1 (*n* = 12) *n* (%)	BIG 2 (*n* = 53) *n* (%)	BIG 3 (*n* = 113) *n* (%)	*p*
Clinical neurological deterioration	0 (0)	0 (0)	9 (8.0)	—
Radiological worsening or new lesion	0 (0)	0 (0)	13 (11.5)	—
Neurosurgical intervention	0 (0)	0 (0)	3 (2.7)	—
ICU admission	0 (0)	0 (0)	7 (6.2)	—
In-hospital mortality	0 (0)	0 (0)	13 (11.5)	—
Composite adverse outcome *	0 (0)	0 (0)	31 (27.4)	<0.001

Note. BIG = Brain Injury Guidelines. * Composite adverse outcome defined as the presence of at least one of the following: clinical neurological deterioration, radiological worsening or new lesion, neurosurgical intervention, ICU admission, or in-hospital mortality. All adverse outcomes occurred exclusively in BIG 3 patients. Dashes (—) indicate formal testing was not applicable due to zero-cell counts. Estimates are based on available in-hospital data; outcomes after transfer were not fully captured.

**Table 5:** Diagnostic performance of the BIG for predicting adverse outcomes.

Metric	Estimate	Interpretation/Remarks
Sensitivity	100%	All patients with adverse outcomes were classified as BIG 3; no false negatives
Negative predictive value (NPV)	100%	No BIG 1 or BIG 2 patient experienced any adverse outcome
Positive predictive value (PPV)	27.40%	31 of 113 BIG 3 patients had at least one adverse outcome
Specificity	Not reliably estimated	High proportion of BIG 3 patients without complete post-transfer outcome data

Note. BIG = Brain Injury Guidelines; NPV = negative predictive value; PPV = positive predictive value. BIG 3 was used as the positive test result; BIG 1 or BIG 2 as the negative test result. Adverse outcome was defined as the composite measure described in Table 4. Specificity and its confidence interval could not be reliably estimated due to the high proportion of BIG 3 patients and incomplete outcome capture after institutional transfer. These results are exploratory and should be interpreted with caution.

## Data Availability

The original data presented in this study are contained within the article. Further inquiries can be directed to the corresponding author.
